# Improved SSA-Based GRU Neural Network for BDS-3 Satellite Clock Bias Forecasting

**DOI:** 10.3390/s24041178

**Published:** 2024-02-11

**Authors:** Hongjie Liu, Feng Liu, Yao Kong, Chaozhong Yang

**Affiliations:** 1 College of Computer Science, Xi’an Polytechnic University, Xi’an 710600, China; hongjieliu@stu.xpu.edu.cn; 2 College of Electronics and Information, Xi’an Polytechnic University, Xi’an 710600, China; kongyao19@xpu.edu.cn; 3 National Time Service Center, Chinese Academy of Sciences, Xi’an 710600, China; ycz@ntsc.ac.cn

**Keywords:** satellite clock bias, GRU neural network, SSA

## Abstract

Satellite clock error is a key factor affecting the positioning accuracy of a global navigation satellite system (GNSS). In this paper, we use a gated recurrent unit (GRU) neural network to construct a satellite clock bias forecasting model for the BDS-3 navigation system. In order to further improve the prediction accuracy and stability of the GRU, this paper proposes a satellite clock bias forecasting model, termed ITSSA-GRU, which combines the improved sparrow search algorithm (SSA) and the GRU, avoiding the problems of GRU’s sensitivity to hyperparameters and its tendency to fall into local optimal solutions. The model improves the initialization population phase of the SSA by introducing iterative chaotic mapping and adopts an iterative update strategy based on t-step optimization to enhance the optimization ability of the SSA. Five models, namely, ITSSA-GRU, SSA-GRU, GRU, LSTM, and GM(1,1), are used to forecast the satellite clock bias data in three different types of orbits of the BDS-3 system: MEO, IGSO, and GEO. The experimental results show that, as compared with the other four models, the ITSSA-GRU model has a stronger generalization ability and forecasting effect in the clock bias forecasting of all three types of satellites. Therefore, the ITSSA-GRU model can provide a new means of improving the accuracy of navigation satellite clock bias forecasting to meet the needs of high-precision positioning.

## 1. Introduction

The basic principle of a GNSS is to use the known position and time information broadcast by satellites for positioning. The known position and time data broadcast by the satellites are specifically represented by the satellite orbit and satellite clock bias data in the navigation messages. Therefore, the accuracy of satellite orbit and clock products affects the positioning accuracy of the GNSS [1,2,3,4,5]. At present, calculation of the final clock bias product data with the highest accuracy is not performed in real-time, instead having a certain time delay [6]. In order to catalyze advancements in GNSS positioning accuracy, it is important to establish a high-precision real-time satellite clock bias forecasting model [7,8,9].

Researchers have established a variety of models for the study of satellite clock bias forecasting, including polynomial models [10], the gray model [11], and the Kalman filter model [12,13]. However, the noise in the quadratic polynomial model is considered an error that obeys a normal distribution. Therefore, as errors in the forecasting process accumulate, the forecasting accuracy decreases over time. The gray model is prone to certain disadvantages when coping with nonlinear problems, which usually make its forecasting accuracy more limited. The prediction effect of the Kalman filter model depends on the degree of knowledge regarding the operating characteristics of the atomic clock and stochastic prior information. Considering the limitations of a single forecasting model, a combined clock bias forecasting model has been proposed [14,15,16]. Experimental findings have indicated that combined models exhibit a moderate enhancement in forecasting accuracy and greater stability in comparison with individual models. However, the combination of multiple models leads to higher complexity, with performance being affected to a certain extent by the individual models involved; moreover, there can be difficulty in selecting optimal weights for individual models when using a combined model [17].

As satellite clocks are susceptible to the influence of the external environment, the presence of noise due to clock bias is unavoidable; as a result, satellite clock bias data exhibit nonlinear characteristics. However, traditional models are insufficient for expressing the nonlinear characteristics of error sources, and further improving the prediction accuracy is difficult [18,19,20,21]. Nevertheless, neural networks demonstrate heightened sensitivity to nonlinear challenges compared to conventional models, offering a means by which to surmount the limitations of traditional approaches and thereby enhance forecast accuracy [22]. In one study [23], a wavelet neural network was employed to forecast mid- and long-term satellite clock bias, revealing superior performance compared to the commonly utilized quadratic polynomial, gray, and Kalman filter models. In another cited work [24], a BP neural network was utilized for satellite clock bias forecasting, with experimental results indicating superior accuracy over the traditional gray model. Notably, the BP neural network exhibited heightened accuracy, especially in the early stages of prediction. Another study [25] proposed an improved model combining spectral analysis and a generalized regression neural network for the BDS-2 satellite. The results indicated that, when compared to the spectral analysis model and the generalized regression neural network model, the improved model outperformed the spectral analysis model and the generalized regression neural network model in terms of convergence speed and prediction accuracy, with improvement noted in all aspects. Another study [26] proposed a nonlinear autoregressive model (NARX) based on external source input. Their experimental results suggested that the accuracy and stability of the model were significantly better than those of traditional models such as the quadratic polynomial model and gray model; in particular, the advantage of this model was obvious when forecasting a cesium atomic clock. A further study [27] proposed a clock bias forecasting model that combined supervised learning and a long short-term memory neural network. The experimental results showed that the model had a significant advantage in controlling the accumulation of the forecasting error over time and that it was suitable for medium- and long-term clock bias forecasting. Another study [28] proposed a BP neural network model for the BDS-3 satellite based on the beetle optimization algorithm. The experimental results showed that the model has very high prediction accuracy for short-term satellite clock bias forecasting. The forecasting result of this model was better when compared with the results for the BP model. The authors of [29] proposed a BP neural network with heterogeneous comprehensive learning and dynamic multi-swarm particle swarm optimization for satellite clock bias forecasting. This model can effectively prevent the BP neural network from becoming trapped in local optima while accelerating its development, and it has good prediction accuracy and stability for satellites with different atomic clock types.

The current research on the use of neural networks for forecasting satellite clock bias mainly uses BPNN or LSTM as the base network for direct prediction or combined modeling with other models. Although BPNN is easy to implement and use, it does not have the ability to capture and manage long-term dependencies in time series. Additionally, although LSTM performs well in time series data prediction, its model structure is more complex. In view of this, the present paper proposes a high-precision clock bias forecasting model, termed ITSSA-GRU. The main contributions of this study are as follows:

- In this paper, a GRU is first applied to the study of satellite clock bias forecasting for the special scenario of satellite clock bias forecasting. GRUs have attracted much attention because of their superior ability to capture and manage long-term dependencies. Compared with LSTM, which is also suitable for time series forecasting, GRUs have a simpler structure and fewer parameters, making them easier to train on relatively small datasets.
- Although GRUs are superior when dealing with long-distance-dependent tasks, they continue to be affected by the limitations of neural networks, namely, that they are prone to falling into local optimal solutions and sensitive to hyperparameters. In order to further improve the accuracy and stability of the GRU for forecasting satellite clock bias data, this paper adopts the SSA to optimize the GRU in order to ensure that the prediction effect of the GRU is not affected by the initial parameters, resulting in a better prediction effect.
- The standard SSA has certain deficiencies in population initialization and individual iteration strategies, leading to the problem of the SSA being prone to falling into local optimal solutions. In order to further improve the forecasting effect of the model, we propose a new algorithm called iterative chaotic map and t-distribution improved SSA (ITSSA), which has better training speed, convergence accuracy, and reliability than the traditional SSA algorithm. Our experimental results show that the ITSSA-GRU model proposed in this paper can be used for satellite clock bias forecasting to effectively improve the accuracy and stability of the forecast.

The rest of this paper is organized as follows: Section 2 introduces the GRU, the SSA, and the basic principles of the ITSSA proposed in this paper; Section 3 presents the construction method of the ITSSA-GRU model, including the preprocessing of clock bias data, the basic structure of the ITSSA-GRU model, and the flow of the algorithm; the experimental results for ITSSA-GRU and four other satellite clock bias forecasting models, namely, SSA-GRU, GRU, LSTM, and GM(1,1), are provided in Section 4, and the results are compared, analyzed, and discussed in detail; finally, our conclusions are presented in Section 5.

## 2. Algorithm Principles

### 2.1. Principle of the GRU Model

GRU is a recurrent neural network model [30] that has been widely used for time-series data forecasting. In contrast to a conventional recurrent neural network (RNN), GRUs adeptly address challenges associated with gradient disappearance and explosion. This efficacy stems from the inherent structural design and characteristics of the backpropagation algorithm. The GRU architecture mitigates the issues commonly encountered in RNNs, enhancing its ability to capture and propagate information across sequential data with improved stability. Compared to long short-term memory (LSTM), GRU simplifies the structure of LSTM by reducing its three gates to two gates, an update gate $Z_{t}$ and reset gate $R_{t}$, thereby greatly improving its calculation speed [31]. This structural simplification enables GRUs to more efficiently process input sequences while maintaining long-term memory [32].

The primary function of the gates $Z_{t}$ and $R_{t}$ is to regulate the flow of information. The inputs to both gates include the current time step input $X_{t}$ and the hidden state from the preceding time step $H_{t-1}$. The calculation formulas for the two gates are as follows:

(1) $$R_{t}=\sigma \left(X_{t}W_{xr}+H_{t-1}W_{hr}+b_{r}\right)$$

(2) $$Z_{t}=\sigma \left(X_{t}W_{xz}+H_{t-1}W_{hz}+b_{z}\right)$$

where $W_{hr}$ and $W_{hz}$ represent the relevant weight matrices and σ represents the sigmoid function. The GRU manages the retention and retrieval of input information through the two gate structures while computing candidate values for the hidden state ${\tilde{H}}_{t}$. The calculation is as shown in Equation (3).

(3) $${\tilde{H}}_{t}=\tanh\left(X_{t}W_{xh}+\left(R_{t}⊙H_{t-1}\right)W_{hh}+b_{h}\right)$$

Finally, it is necessary to update the final state $H_{t}$, which forgets some information from $H_{t-1}$ passed down through the network and adds some information entered by the current node, as shown in Equation (4).

(4) $$H_{t}=Z_{t}⊙H_{t-1}+\left(1-Z_{t}\right)⊙{\tilde{H}}_{t}$$

### 2.2. Sparrow Search Algorithm

The training phase of a neural network model frequently encounters difficulty in attaining a locally optimal solution [33]. To enhance the effectiveness of training, mitigate the risk of falling into local minima, and bolster the model’s robustness, an intelligent optimization algorithm can be employed to refine the initial parameters of the neural network. This approach is anticipated to contribute to enhanced forecasting of BDS clock bias data.

The SSA is a nascent optimization algorithm rooted in swarm intelligence principles [34] that learns from the behavioral strategies of sparrows, including foraging and anti-predation. The authors of [35] compared the performance of four emerging intelligent optimization algorithms: Grey Wolf optimization (GWO), particle swarm optimization (PSO), the differential evolution (DE) algorithm, and the SSA. Their experimental results indicated that the SSA exhibits strong local search ability under a variety of test function experiments, and has the advantages of high precision and fast convergence speed.

The SSA is based on two key behavioral strategies, namely, the producer and scrounger strategies. Individuals in a population observe each other’s behavior. Scroungers compete for food resources to improve their predation efficiency. When sensing danger, a population of sparrows exhibits anti-predation behavior. This intelligent optimization algorithm based on the behavior of sparrows not only performs well in terms of local search ability, it has fast global convergence speed and a high degree of parallelism, providing an effective and innovative method for solving the neural network model optimization problem [36].

The positions of the producers are updated as follows:

(5) $$X_{i,j}^{t+1}=\left\{\begin{array}{c} X_{i,j}^{t}\cdot \exp\left(-\frac{i}{\alpha \cdot iter_{\max}}\right),R_{2}<ST\\ X_{i,j}^{t}+Q\cdot L,R_{2}\geq ST \end{array}\right\}$$

where *t* denotes the current iteration, $iter_{max}$ represents the maximum number of iterations, $X_{i,j}$ represents the first *i* sparrows in the $j^{th}$ dimension, α is a random number between 0 and 1, $R_{2}$ is the early warning value, with a value between 0 and 1, ST is a safe value in the interval between 0.5 and 1, *Q* is a random number that obeys a normal distribution, and *L* is a 1×d-dimensional matrix.

For scroungers,

(6) $$X_{i,j}^{t+1}=\left\{\begin{array}{c} Q\cdot \exp\left(-\frac{X_{worst}-X_{i,j}^{t}}{i^{2}}\right),i>\frac{n}{2}\\ X_{P}^{t+1}+\left|X_{i,j}^{t}-X_{P}^{t+1}\right|\cdot A^{+}\cdot L,i\leq \frac{n}{2} \end{array}\right\},$$

where $X_{p}$ denotes a group of sparrows; the optimal position of the producer is $X_{worst}$, i.e., the worst position in the current sparrow population, while *A* is a 1×d-dimensional matrix with elements randomly set to 1 or –1, where $A^{+}=A^{T}{\left(AA^{T}\right)}^{-1}$. When $i>\frac{n}{2}$, the $i^{th}$ scrounger with a low fitness value in the sparrow population is in a state of foraging in other places.

The initial position of a sparrow that is aware of danger is randomly generated in the population; its mathematical expression is as follows:

(7) $$X_{i,j}^{t+1}=\left\{\begin{array}{c} X_{best}^{t}+\beta \cdot \left|X_{i,j}^{t}-X_{best}^{t}\right|,f_{i}>f_{g}\\ X_{i,j}^{t}+K\cdot \left(\frac{\left|X_{i,j}^{t}-X_{worst}^{t}\right|}{(f_{i}-f_{w})+\varepsilon }\right),f_{i}=f_{g} \end{array}\right\}$$

where $X_{best}$ is the current global optimal position in the sparrow population, β is a parameter used to control the step size, *K* is a random number, $f_{i}$ is the fitness value of the current sparrow individual, and $f_{g}$ and $f_{w}$ are the global optimal and worst fitness values in the current sparrow population, respectively. In addition, a constant term ε is used to avoid the situation where the denominator is zero.

### 2.3. Improvement of the SSA

Compared with other swarm intelligence algorithms, the basic SSA has higher solution accuracy and efficiency along with the advantages of good robustness and stability. Despite these strengths, certain limitations hinder its performance. First, the random initialization of the population during the early stages impedes the attainment of optimal ergodicity. Second, the SSA experiences a decline in population diversity as the number of iterations increases, making it susceptible to entrapment in local optima. To overcome these challenges, we introduce an innovative approach termed the iterative chaotic map and t-step optimization-based SSA.

#### 2.3.1. Iterative Chaos Map

The traditional SSA usually generates an initial population randomly in the search space, which causes an uneven distribution of the sparrow population and has a negative impact on the optimization of subsequent iterative algorithms. To solve this problem, researchers have introduced chaos theory [37,38]. As a nonlinear phenomenon, chaos has the characteristics of ergodicity, randomness, sensitivity, and regularity. The ergodic nature and randomness of chaos make it less likely that the search process will reach a local minimum. Therefore, initializing the positions of individual sparrows by applying a chaotic map is an effective strategy.

Common chaotic maps include the ICMIC map, cubic map, sinusoidal map, sine map, Bernoulli map, and iterative map [39,40,41]. Among these maps, the iterative map has exhibited better robustness and chaos ergodicity in satellite clock bias forecasting experiments compared to other chaotic maps. Employing an iterative chaotic map for refinement of the initial population within the SSA results in more effective dispersion of the algorithm across the search space, which helps to improve the global optimization performance of the algorithm and reduces the possibility of reaching a local minimum, thereby enhancing the robustness of the algorithm [42].

The introduction of this improved method makes the SSA more flexible and reliable in dealing with complex problems. Through the introduction of a chaotic map, the initial state of the algorithm becomes more diverse, providing a broader search space for subsequent optimization iterations. The mapping iteration formula is as follows:

(8) $$X_{t+1}=\sin\left(\frac{b\pi }{x_{t}}\right)$$

where *b* is randomly generated between 0 and 1 and $x_{t}$ is the value of *x* in the $t^{th}$ iteration.

#### 2.3.2. T-Step Optimization

The t-distribution is used to optimize the update strategy in the individual iteration of the SSA. For an endangered individual, we determine whether its fitness is less than the population’s average fitness. If the fitness of an individual is less than the average fitness, indicating that the individual may be in a poor position, then the t-distribution mutation is applied to the position of the individual. This approach generates new locations, introducing randomness and variety. The mutation process is as follows:

(9) $$x_{i}^{t}=x_{i}+x_{i}\cdot t\left(iter\right)$$

where ${x_{i}}^{t}$ is the position after mutation, $x_{i}$ is the original position of the individual, and t(iter) is subject to the t-distribution, with the number of iterations as the degrees of freedom.

The parameter *t*, representing the number of iterations throughout the entire algorithm, serves as the degree-of-freedom parameter for the t-distribution. In the initial stages of the algorithm, the t-distribution is employed to enhance global search capabilities. As the iteration count rises, the distribution gradually shifts towards a Gaussian distribution to strengthen local searching. This dynamic strategy enhances the adaptability of the SSA, significantly improving convergence speed and accuracy. Moreover, it increases the algorithm’s flexibility in addressing diverse search requirements, thereby enhancing its overall performance and efficiency

## 3. Construction of the ITSSA-GRU Clock Bias Forecasting Model

### 3.1. Data Source and Pre-Processing

**Figure 1:**
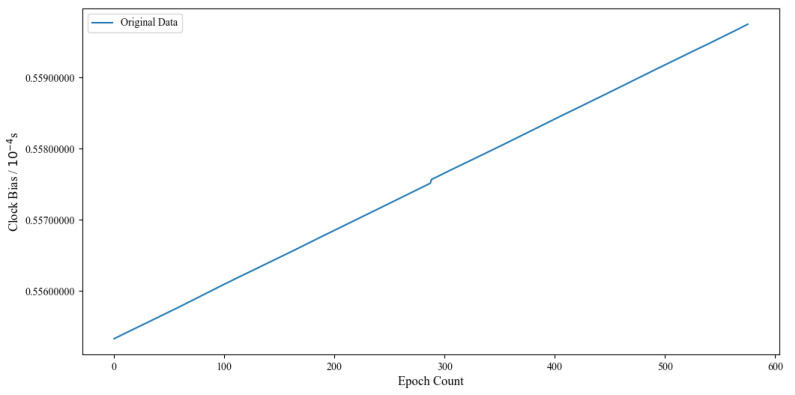
Original C38 clock bias data.

In this study, we used precision clock bias data from the BDS-3 satellites with a 5 min interval that were provided by iGMAS for experimental analysis. Satellite clock bias data are usually presented in the form of phase data. Taking the C38 satellite clock bias data from 31 January 2023 00:00 to 1 February 2023 00:00 (UTC) as an example, Figure 1 shows the original clock bias with a time interval of 5 min. With respect to phase data, the original clock bias data usually exhibit a linear trend between different epochs, while the variations in the clock bias data between adjacent epochs are relatively small.

Neural network models perform well when dealing with non-linear data, and have excellent fitting ability and generalization ability; therefore, to make these clock bias data suitable for the nonlinear expression of neural networks, the authors of [43] adopted the strategy of differentially processing the original clock bias data and converting them into frequency data, making the data more suitable for processing by neural networks. This processing strategy helps to better capture the complex nonlinear characteristics of clock bias data, improving the performance and accuracy of the neural network model. In particular,

(10) $$y_{i}=\frac{x_{i+1}-x_{i}}{\Delta t},$$

where $x_{i+1}$ and $x_{i}$ are the t+1 and *t* phase values, respectively, and Δt is the time interval between adjacent epochs.

**Figure 2:**
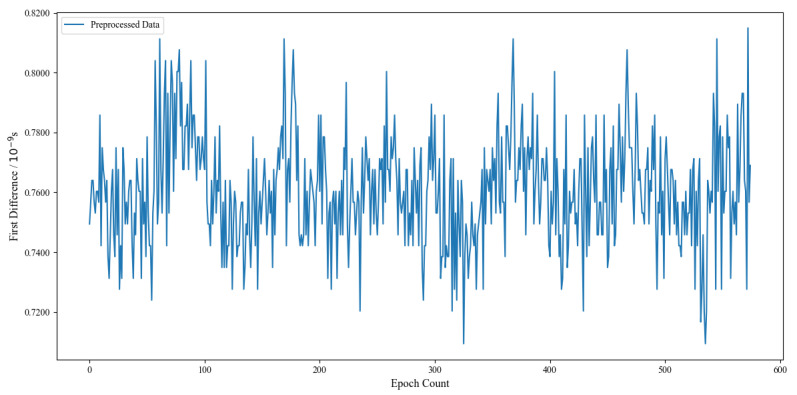
C38 clock bias data after preprocessing.

In the operational phase of a spaceborne atomic clock, there are instances where the volume of satellite clock bias data may experience fluctuations. In pursuit of further enhancing the forecasting accuracy of satellite clock bias data, it is necessary to process the data using an outlier detection methodology. This method involves converting the original clock bias data from phase data to frequency data and subsequently employing the Median Absolute Deviation (MAD) to detect outliers. In this method, the first differential data are combined into $\Delta l\left(i\right)$. If the following condition is met

(11) $$|\Delta l\left(i\right)|>\left(k+n*MAD\right),$$

then the first difference data are considered to be outliers and should be excluded. Moreover, we use the interpolation method to supplement the excluded data. In Equation (11), $MAD=Median\left\{|\Delta l\left(i\right)-k|/0.6745\right\}$ and $k=Median\left\{\Delta l\left(i\right)\right\}$. The processed clock bias frequency data are shown in Figure 2.

The first difference method and the outlier detection method were used to preprocess the data in order to obtain the difference series. The model uses the idea of a sliding window to forecast the clock bias in a step-by-step manner, continuously replacing the previously known data with new forecast data. Supposing that we have a set of clock bias data $\left\{a_{1},a_{2},\ldots ,a_{t}\right\}$, the training dataset can be constructed as $\left\{\left[\left(a_{1},a_{2},\ldots ,a_{m}\right),\left(a_{m+1}\right)\right],\left[\left(a_{2},a_{3},\ldots ,a_{m+1}\right),\left(a_{m+2}\right)\right],\ldots ,\left[\left(a_{t-m},a_{t-m+1},\ldots ,a_{t-1}\right),\left(a_{t}\right)\right]\right\}$. The training dataset contains t−m groups of training data, each of which is composed of input samples and output samples.

### 3.2. ITSSA-GRU Clock Bias Forecasting Model

In the training phase of the GRU, the choice of initial weights and thresholds plays a crucial role, exerting a considerable impact on the network’s convergence speed and accuracy. Random initialization of parameters is likely to cause the training to become stuck in a local optimal solution, which becomes a bottleneck limiting the prediction performance of the model.

**Figure 3:**
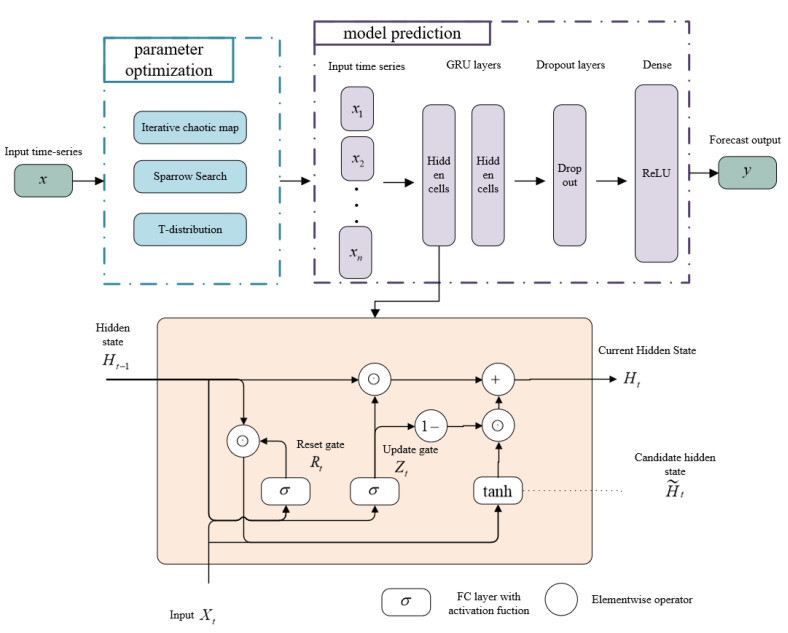
Structural diagram of the ITSSA-GRU model.

To overcome this problem, the proposed ITSSA-GRU model incorporates an enhanced SSA with augmented search capabilities. This algorithm is utilized to optimize the initial weights and thresholds of the GRU. The augmentation of the SSA aims to strengthen its global search capability by automating the adjustment of neural network parameters to minimize human intervention. This automated process not only diminishes the need for extensive parameter tuning, it improves the training efficiency of the model, thereby reducing the risk of the model becoming trapped in local minima during the training process. This strategic approach is expected to yield superior solutions, resulting in enhanced model generalization and improved forecasting performance through optimization of the initial weights and thresholds. Figure 3 shows the structural diagram of the ITSSA-GRU model.

The structure of the ITSSA-GRU model consists of two main components, namely, parameter optimization and GRU forecasting. In the initial segment, the ITSSA algorithm optimizes the initial weights and thresholds of the GRU. This process begins by utilizing the iterative chaos map to optimize the initial population of the SSA. The SSA is then employed to calculate the positional information of the sparrows, and the iterative update strategy is ultimately refined using the t-distribution. In the subsequent segment, the optimized parameter information is fed into the GRU for satellite clock bias forecasting. The model comprises two GRU layers, namely, a dropout layer and a fully connected layer. To enhance the model’s expressive capacity, the ReLU activation function is applied to the fully connected layer. Throughout the network training stage, the Adam algorithm (an adaptive optimization algorithm) collaborates with the GRU neurons to train on the input data, thereby enhancing the training efficiency and overall network performance. In this study, we used a modified SSA to automatically optimize the parameters of the GRU network in order to avoid the problem of manual setting of the parameters leading to the model falling into local optimal solutions. The relevant parameters of the SSA along with the upper and lower thresholds for each parameter of the GRU network were determined during the experiment.

The specific parameters and threshold settings used during the experiment are shown in Table 1.

The number of producers in the sparrow population was set to 20% of the total number of individuals, while the number of vigilantes was set to 10% of the total number of individuals. The maximum number of iterations for the search was set to 50. For the hidden layer of the GRU network, the search threshold for the number of hidden units was limited to a number between 10 and 40. The threshold for the maximum number of iterations was then set between 1000 and 2000. In addition, the threshold for the learning rate was set in the range of 0.01 to 0.1. This setting was intended to maintain the effectiveness of the algorithm and limit the breadth of the search when exploring the parameter space in order to ensure that a suitable network configuration is found. These thresholds were chosen based on the nature of the research problem, the availability of computational resources, and empirical adjustments to provide a fundamental guarantee of the experiments’ robustness and reproducibility.

**Figure 4:**
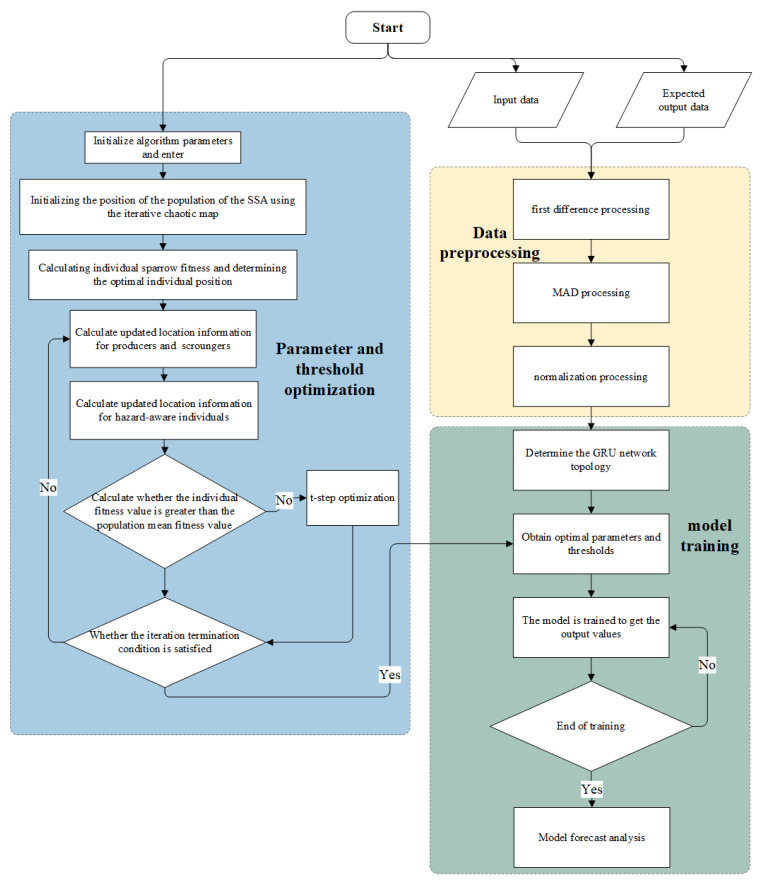
Flowchart of the ITSSA-GRU algorithm.

Figure 4 shows the flow chart for the ITSSA-GRU algorithm.

The procedure for satellite clock bias forecasting using the proposed ITSSA-GRU model is outlined as follows:

(1) Initialization of the parameters for the GRU, encompassing the learning rate, loss function, and maximum number of training iterations.
(2) The first difference method and median deviation method are used to process the clock bias data, then the normalization method is used to normalize the data to the interval [−1, 1].
(3) After preprocessing, the clock bias data are segmented into input and expected output sections utilizing a sliding window approach.
(4) The initial weights and thresholds of the GRU are optimized using the improved SSA (ITSSA); specifically, the ITSSA optimizes the parameters of the GRU network as follows:
  a. Initialize all the parameters of the ITSSA algorithm, including the population size, search space range, number of iterations, optimization objective, and chaotic map parameters.
  b. Initialize the position of the population for the SSA using the iterative chaotic map method.
  c. Evaluate the fitness of each individual sparrow and identify the optimal individual positions.
  d. Calculate the current position of each individual sparrow, including the producers and scroungers.
  e. Compute the revised positional information for the individuals with awareness of potential hazards.
  f. Evaluate individuals considered at risk by comparing their fitness to the population average. If an individual’s fitness is below the average, suggesting a potentially unfavorable position, apply the t-distribution mutation to adjust the individual’s position. This method introduces randomness and diversity, generating new locations.
  g. If the algorithm reaches the maximum iteration limit, the parameter optimization process concludes; otherwise, the algorithm returns to step d until the specified criteria are satisfied. Upon completion of the iteration, the initial weights and thresholds for the neural network are acquired.
(5) The GRU neural network, now equipped with the optimized initial weights and thresholds, undergoes training on the sample dataset generated in Step (3).
(6) The trained network is employed for data forecasting, and the predicted values undergo the normalized inverse operation and the inverse operation of the first difference method. These operations yield the final forecast satellite clock bias data.

## 4. Experiments and Results

To assess the predictive performance of the ITSSA-GRU model on BDS-3 clock bias data, experiments were conducted using data from 31 January 2023 00:00 to 1 February 2023 00:00 (UTC). The future clock bias of the two types of satellites, namely, MEO and IGSO, were forecast for 3 h, 6 h, 12 h, and 24 h. Considering the possibility that different types of orbits as well as different types of onboard atomic clocks may have an impact on the forecasting effect, two satellites with three different types of orbits, namely, BDS-3 MEO, IGSO, and GEO, were randomly selected for our experiments in order to validate the generalization ability and robustness of the model. The root mean square error (RMSE), which is the average of the squares of the differences between the predicted values and the actual values, was used as the statistical measure of forecasting accuracy; the smaller the RMSE is, the higher the forecasting accuracy. Its formula is as follows:

(12) $$RMSE=\sqrt{\frac{1}{N}\sum_{i=1}^{N}{\left(y_{i}-\hat{y_{i}}\right)}^{2}}$$

where *N* denotes the amount of data, $y_{i}$ denotes the actual clock bias, and $\hat{y_{i}}$ denotes the forecasted value. The absolute value of the difference between the maximum error and the minimum error (Range) was used as the statistical measure of forecast stability. In order to analyze the forecasting effect of the ITSSA-GRU model, the LSTM model, GRU model, SSA-GRU model, ITSSA-GRU model, and GM model were used to forecast the clock bias data of the BDS.

In order to further evaluate the forecasting performance of the model, this paper uses the cumulative distribution function (CDF) plot of the errors and box plots to express the distribution of the forecasting errors. The CDF plot mainly shows the performance of the model as a proportion of the overall sample at different error levels. It is able to show the distribution over the entire error range as well as the performance of the model at small error levels. The error box plot provides an overall overview of the error distribution, including the median, upper, and lower quartiles and outliers. It places more emphasis on the overall distribution and dispersion of the error and possible outliers.

### 4.1. Forecast Results for the BDS-3 MEO Satellite

**Figure 5:**
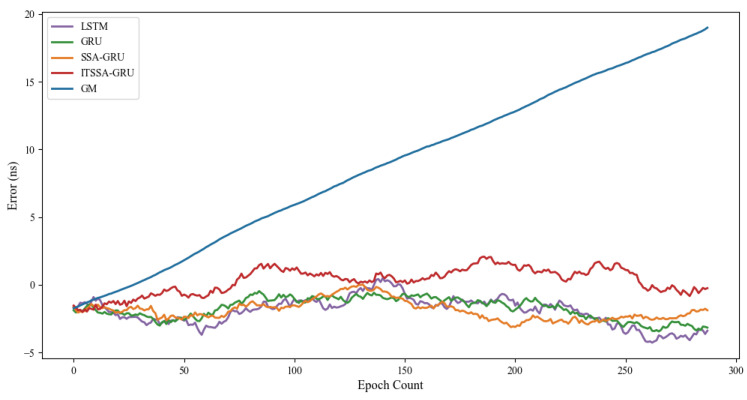
C36 satellite clock bias forecast.

**Figure 6:**
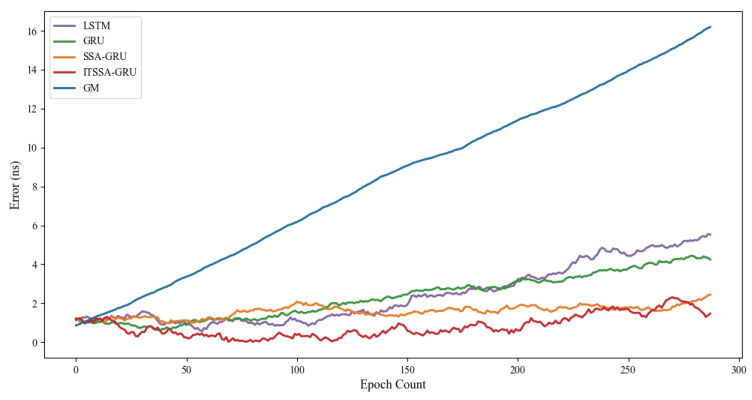
C37 satellite clock bias forecast.

**Figure 7:**
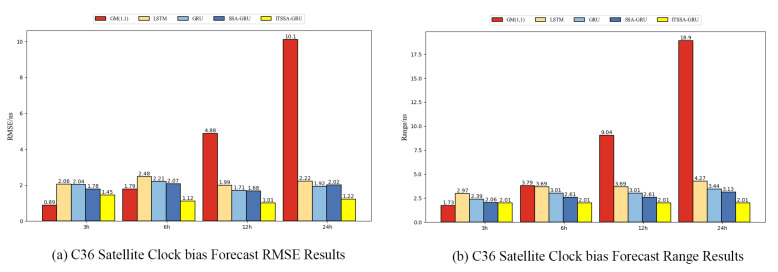
Histograms of the RMSE and range at each stage for the C36 satellite.

**Figure 8:**
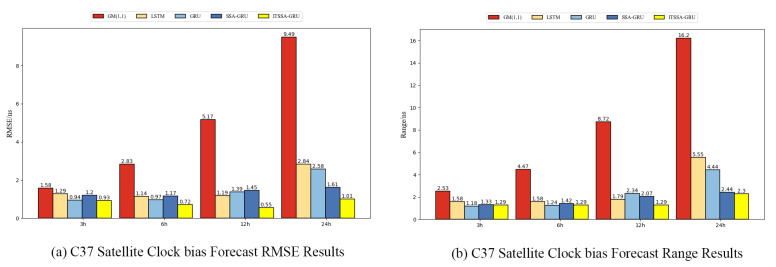
Histograms of the RMSE and range at each stage for the C37 satellite.

For the experiment, two BDS-3 MEO satellites, C36 and C37, were chosen. The atomic clocks carried by these two satellites are rubidium atomic clocks (Rb-II); the error curves of the prediction experiments are shown in Figure 5 and Figure 6. The histograms of the RMSE and range of the two satellites in the four prediction phases of C36 and C37, namely, 3 h, 6 h, 12 h, and 24 h, are shown in Figure 7 and Figure 8, respectively. Table 2 demonstrates the percentage improvement in the RMSE and range of the ITSSA-GRU model compared to the other four models. The experimental results show that the ITSSA-GRU model is significantly better than the other four models for 3 h, 6 h, 12 h, and 24 h clock bias forecasts of MEO satellites, especially in the middle and late stages of the forecast. The results for the LSTM and GRU models are significantly worse than those for the SSA-GRU and ITSSA-GRU models optimized using the SSA in the late stages of the forecast. The error of the GM(1,1) model, on the other hand, accumulates with the increase in forecasting time and basically increases with a linear trend; the overall forecasting effect is obviously inferior to that of the other four models, with the errors finally reaching 18.9 ns and 16.19 ns, respectively.

For the C36 satellite, in the early stage of forecasting the GM(1,1) model has higher forecasting accuracy compared to the other four models, with a maximum error of only 1.73 ns during the first 3 h forecasting phase. The forecasting levels of the LSTM and GRU models are similar, while the forecasting performance of the SSA-GRU model after optimization using the SSA is better than that of the LSTM and GRU models. After improvement using the SSA, the ITSSA-GRU model further improves the forecasting accuracy of the SSA-GRU model in the early stage of forecasting. In the mid-term forecast, the forecasting error of GM(1,1) increases continuously, and the forecasting accuracy is obviously insufficient. While the other four models have some improvement in accuracy, the forecast error range for the ITSSA-GRU model stabilizes near 0 ns for longer durations, which indicates more stable and more effective forecasting compared with the other three neural network models. In the later stage of forecasting, all four neural network models experienced increasing errors with the accumulation of model errors. However, for the parameter-optimized SSA-GRU and ITSSA-GRU models, the error range remains the same after the mid-term increase in forecast error. Again, further continuous expansion can improve forecasting accuracy and reduce the associated error, and the effect is better when compared with the other two neural network models. The improvement in accuracy of the ITSSA-GRU model is more obvious in the later stage, with the final local error maintained between 0 and 1 and the global maximum error stable at approximately 2 ns.

For the C37 satellite, although the initial forecast error of the GM(1,1) is small, the growth rate of the error in the subsequent process is obvious and the forecast effect in all four stages is poor. Nevertheless the advantages of the ITSSA-GRU model become more pronounced in terms of both accuracy and stability within the 24-hour forecasting period. The forecasting accuracy and stability of the ITSSA-GRU model proves to be more reliable than those of the other four models. In the initial forecasting stages, the GRU model exhibits slightly better accuracy than the LSTM and SSA-GRU models. However, with the progression of forecasting time the accuracy of the LSTM and GRU models is significantly diminished due to the accumulation of errors. Although the SSA-GRU model experiences lower accuracy in the early stages due to high model complexity, after SSA optimization its forecasting results become more stable in the middle and late stages without substantial increases in error. In comparison with the other four models, the ITSSA-GRU model demonstrates a noteworthy improvement in forecasting accuracy in the early stages, fixing the SSA-GRU model’s lack of accuracy in the pre-forecasting period. Although the forecasting accuracy of the ITSSA-GRU model slightly decreases with increasing forecast length, the increase in the error is small and the final forecasting error stabilizes at about 2 ns.

**Figure 9:**
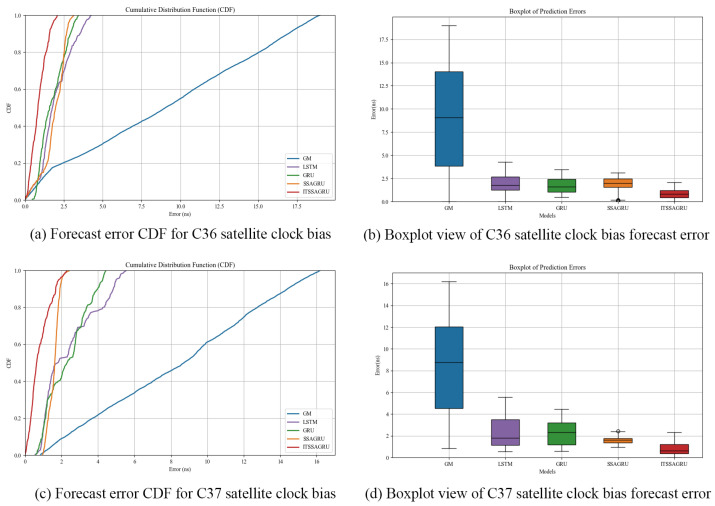
CDF and box plot analysis of satellite clock bias forecast for C36 and C37 satellites.

Figure 9 shows the forecast error CDF plots and box plots for the C36 and C37 satellites. The error CDF plots of the two satellites are illustrated in Figure 9a,c, from which it can be seen that the CDF curve of the gray model rises slowly, indicating that the model has relatively poor prediction results in small error ranges along with a large error range. On the contrary, the ITSSA-GRU model shows a significantly steeper rising trend and has the smallest error range, indicating more accurate prediction. This highlights the robustness of the ITSSA-GRU model in realizing highly accurate predictions in our clock bias forecast experiments for the C36 and C37 satellites. In Figure 9b, the box plots for the bell bias forecast for the C36 satellite are illustrated; it can be seen that the ITSSA-GRU model has the shortest box, indicating the highest concentration of data, which suggests more stable forecasting performance. Figure 9d shows the error box plot for the C37 satellite; it can be seen that the box of the SSA-GRU model is the shortest and that the median, upper, and lower quartiles of the error of the SSA-GRU model are lower relative to other models except for the ITSSA-GRU model, which indicates that direct parameter optimization of the GRU using the SSA achieved certain results for the C37 satellite experiments. The forecasting accuracy and stability are both improved. Although the concentration of the error of the ITSSA-GRU model is not as good as that of the SSA-GRU, its forecasting accuracy is higher and the median, upper, and lower quartiles of the error are lower, close to 0 ns.

### 4.2. Forecast Results for the BDS-3 IGSO Satellite

**Figure 10:**
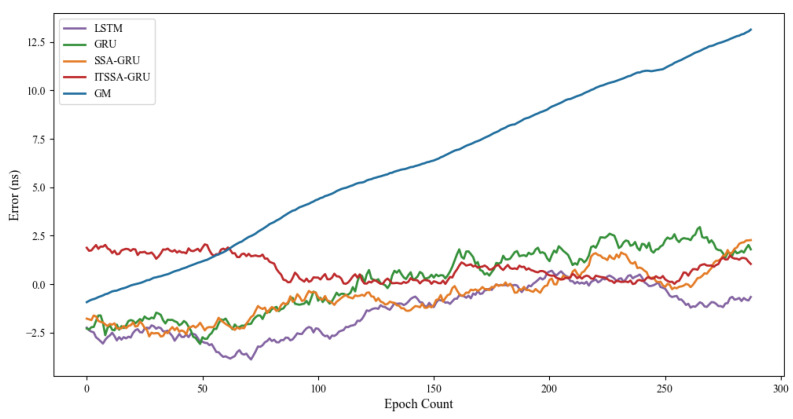
C38 satellite clock bias forecast.

**Figure 11:**
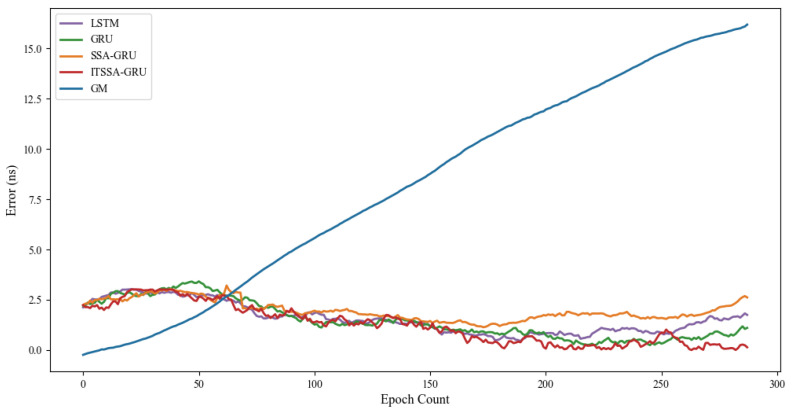
C40 satellite clock bias forecast.

**Figure 12:**
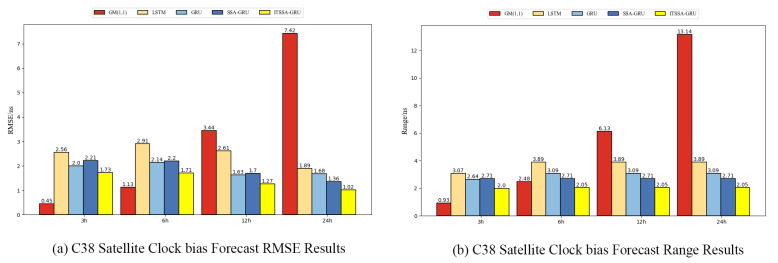
Histograms of the RMSE and range at each stage for the C38 satellite.

**Figure 13:**
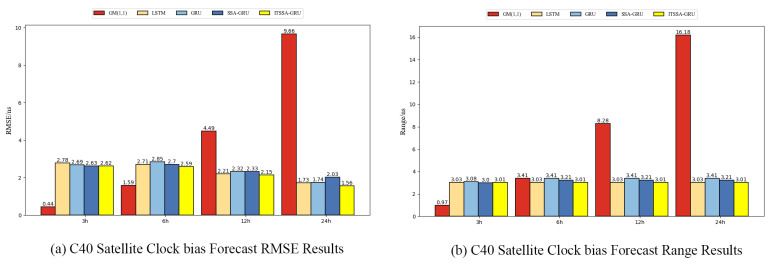
Histograms of the RMSE and range at each stage for the C40 satellite.

Two BDS-3 IGSO satellites, C38 and C40, were selected for this experiment; the atomic clocks carried by these two satellites are both hydrogen atomic clocks (PHM). The error curves of the forecasting experiments are shown in Figure 10 and Figure 11. The histograms of the RMSE and range of the C38 and C40 satellites for the four forecast phases, namely, 3 h, 6 h, 12 h, and 24 h, are shown in Figure 12 and Figure 13. Table 3 demonstrates the percentage improvement in the RMSE and range of the ITSSA-GRU model compared to the other four models. The experimental results show that the ITSSA-GRU model outperforms the other four models for 3 h, 6 h, 12 h, and 24 h clock bias forecasts for IGSO satellites. Using the ITSSA-GRU model to forecast the clock bias for the C38 and C40 satellites, it can be seen from the trend graph that although the effect is better than the other three neural network models in the early stage of the forecast, the overall forecast accuracy is not high. With increasing forecasting time, the ITSSA-GRU model gradually becomes more accurate and more stable in the middle and late stages. As for the GM(1,1) model, its prediction in the experiments with the two IGSO satellites is better in the early stage, and has a lower initial prediction error compared with the other four models, with the initial error basically remaining near 0 ns. However, the error continues to increase afterwards, and in the forecasts after 6 h the error exceeds the other four models and maintains its increasing trend, finally reaching about 12 ns and 15 ns, respectively, for the two satellites.

For the C38 satellite, the forecast error of the ITSSA-GRU model in the early stage of forecasting is approximately between 1 and 2 ns, and there is no obvious fluctuation in the error. The forecasting errors of the other three neural network models are approximately between 1.5 and 3 ns. The GRU, LSTM, and SSA-GRU models experience significant fluctuations in the error during the early stage of forecasting, with the forecasting errors of the GRU and SSA-GRU models even reaching approximately 3 ns. The LSTM forecasting results are even worse, with the forecasting error reaching approximately 4 ns. In the mid-term forecast, the forecasting accuracy of the four neural network models all increase; the error of the ITSSA-GRU model is basically controlled between 0 and 1 ns, while the other three neural network models have larger error margins and their forecasting accuracy is obviously insufficient. In the later stage of the forecast, while the forecasting errors of the four neural network models all increase with the accumulation of the error, the forecasting error of the ITSSA-GRU model remains at approximately 1 ns, which is smaller than the errors of the other three neural network models, indicating better forecasting results. In addition, the forecast results for ITSSA-GRU are very stable, with the maximum forecasting error controlled at approximately 2 ns within the 24 h global forecasting range.

For the C40 satellite, the forecast accuracy of the ITSSA-GRU model was not significantly improved compared to those of the other three neural network models in the early stage of the forecast. It can be seen from the trend chart and the forecast error statistics table that the forecast results of the four neural network models in the early stage of forecasting are very similar. The accuracy and stability of the forecasts are almost the same, and the advantage of ITSSA-GRU is not obvious. In the mid-term forecast, the predictive performance of the four neural network models all showed improvement. The ITSSA-GRU model demonstrated certain advantages compared to the other three neural network models, albeit with only modest enhancements in forecast accuracy. During the 6-hour forecast period, ITSSA-GRU achieved improvements in accuracy of 4.43%, 9.12%, and 4.07% compared to LSTM, GRU, and SSA-GRU, respectively. Similarly, during the 12-hour forecast period, ITSSA-GRU showed enhancements in accuracy of 2.71%, 7.33%, and 7.73%, respectively. In the later stage of the forecast, the forecasting errors of the LSTM and SSA-GRU models increase significantly, while the results for the GRU and ITSSA-GRU models are more stable, the forecasting accuracy is significantly greater, and there is no significant fluctuation in the error. ITSSA-GRU has a smaller mean error range and higher forecast accuracy compared to GRU; at 24 h, the forecasting error for GRU remains between 1 and 1.5 ns, while the ITSSA-GRU error remains stable between 0 and 0.5 ns. In general, the forecasting level of the ITSSA-GRU model yields better results than the other four models, with significant improvements in both accuracy and stability.

**Figure 14:**
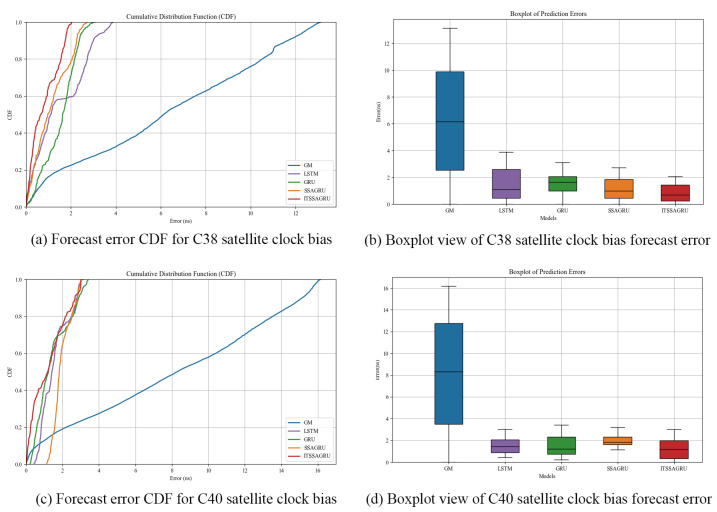
CDF and box plot analysis of satellite clock bias forecast for satellites C38 and C40.

Figure 14 shows the CDF plots and box plots of the forecasting error for the C38 and C40 satellites. The error CDF plots for the two satellites are illustrated in Figure 14a,c; it can be seen that the trend of the CDF plots for the ITSSA-GRU model is steeper compared to the trend of all the other models. Among them, for the C38 satellite, the forecasting accuracy of ITSSA-GRU is more obvious and the error is kept within 2 ns, indicating better forecasting accuracy. For the C40 satellite, it can be seen from the figure that except for the gray model, where the CDF trend is obviously flat, the steepness of the other four models is closer, especially in the region where the error is between 2 and 4 ns, indicating that the models maintain consistent performance for moderate errors. Although the steepness of the model in the range of errors from 0 to 2 ns indicates that the model’s forecast performance is relatively good within this range, the model has a higher number of individuals for which the forecast error remains within 2 ns. Figure 14b,d show the error box plots for the two satellites; from these, it can be seen that neither of the boxes for the ITSSA-GRU model is the shortest, indicating that the distribution of the model’s forecasting errors is not concentrated. The median and quartiles are relatively well placed, indicating the accuracy of the model for most of the samples.

### 4.3. Forecast Results for the BDS-3 GEO Satellite

**Figure 15:**
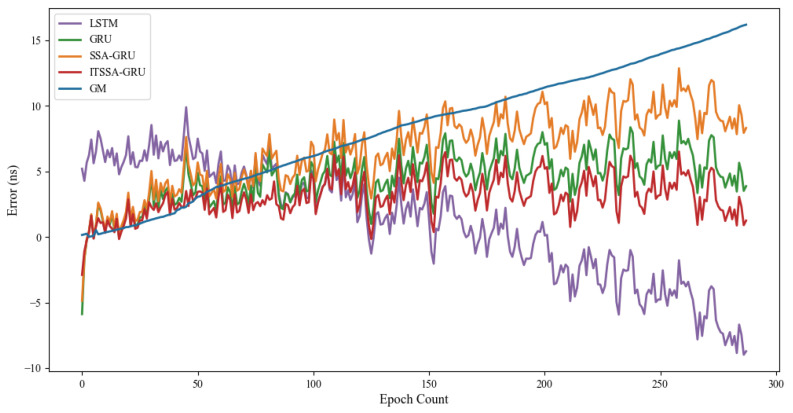
C59 satellite clock bias forecast.

**Figure 16:**
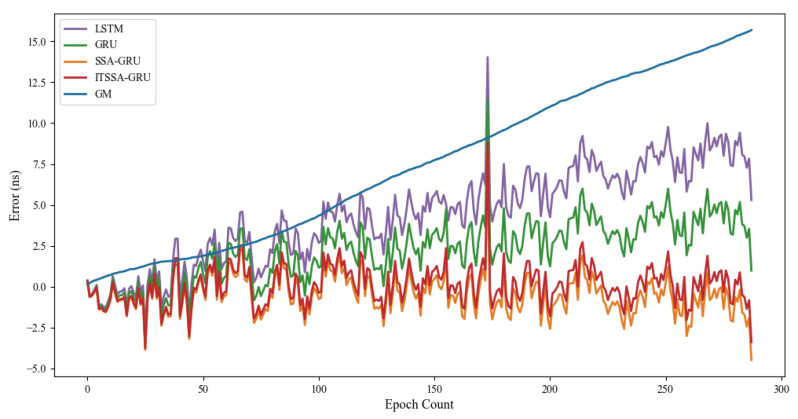
C60 satellite clock bias forecast.

**Figure 17:**
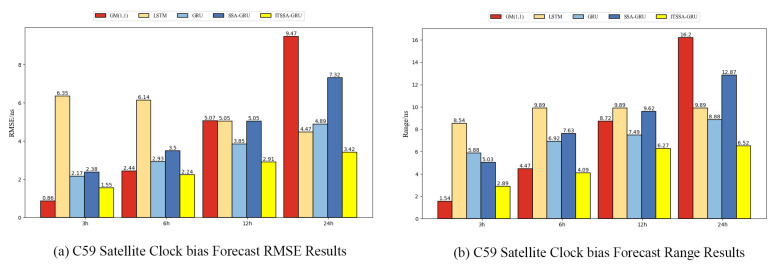
Histograms of the RMSE and Range at each stage for the C59 satellite.

**Figure 18:**
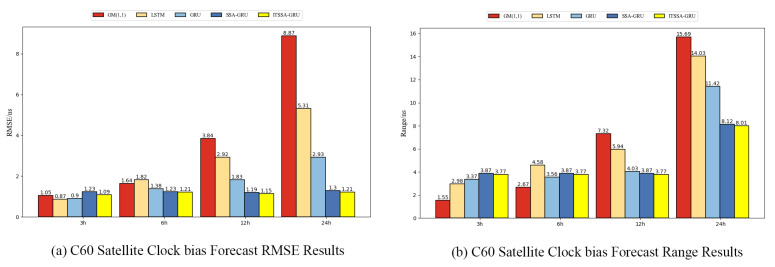
Histograms of the RMSE and range at each stage for the C60 satellite.

In order to further verify the generalization ability of the ITSSA-GRU model, we additionally selected the data from 15 February 2023, 00:00 to 16 February 2023, 00:00 (UTC) for modeling and conducted forecasting experiments for two BDS-3 GEO satellites, C59 and C60, which carry atomic clocks consisting of hydrogen atomic clocks. The errors of the forecasting experiment curves are shown in Figure 15 and Figure 16. The histograms of the RMSE and range for the two satellites are shown in Figure 17 and Figure 18 for the four forecasting phases of 3 h, 6 h, 12 h, and 24 h, while Table 4 presents the percentage improvement in the RMSE and range of the ITSSA-GRU model compared to the other four models. The experimental results show that the four neural network models used in the forecasting experiments for these two GEO satellites have larger forecasting errors and poorer forecasting stability than those for the MEO and IGSO types of satellites. The maximum errors of the LSTM, GRU, SSA-GRU, and ITSSA-GRU models for satellite C59 reach 9.89 ns, 8.88 ns, 12.87 ns, and 6.52 ns, respectively, while the maximum errors of the four models for satellite C60 reach 14.03 ns, 11.42 ns, 8.12 ns, and 8.01 ns, respectively. The forecasting effect of the GM(1,1) model is comparable to that observed in the experiments on the MEO and IGSO types of satellites, and continues to maintain a low initial error. However, the cumulative error becomes larger with increasing forecast length; this effect becomes poorer in the middle and late stages of the forecast, and there is a large gap between GM(1,1) and the other four models. The highest errors in the forecasting experiments with satellites C59 and C60 are 16.2 ns and 15.69 ns, respectively. Although the overall forecasting effect is poor due to the characteristics of GEO satellites, the ITSSA-GRU model continues to have advantages over the other four models, and the fluctuation of the forecasting error is relatively stable. Especially in the middle and late stages of forecasting, the range of error fluctuation does not increase significantly.

For satellite C59, the error of the GM(1,1) model is better during the pre-forecast period, and the forecasting error is smoother compared to those of the other four models. The other four models have larger forecasting errors in the early stage; the initial error values of the LSTM, GRU, SSA-GRU, and ITSSA-GRU models are 8.54 ns, 5.88 ns, 5.03 ns, and 2.89 ns, respectively. The ITSSA-GRU model reduces the problem of larger initial errors to a certain extent, and although the effect is not as good as that of the GM(1,1), the initial forecast errors are lower than those of the other three models. In the middle and late stages, the forecasting errors of the LSTM, GRU, and SSA-GRU models have obvious fluctuations, while the ITSSA-GRU model is smoother; moreover, it has a smaller error range, and the maximum error is controlled below 7 ns. The resulting forecasts are improved by 63.89%, 23.49%, 30.06%, and 53.28% for the 24 h forecasting time compared to those of the other four models.

For the C60 satellite, the optimized SSA-GRU and ITSSA-GRU models do not show effective improvements in accuracy compared with the other three single models in the early forecast stage. However, the advantages of these two models gradually become more apparent in the forecast stages after 3 h, especially during the forecast stage after 6 h, where the errors of the other three single models obviously increase while the error curves of the SSA-GRU and ITSSA-GRU remain relatively smooth without significant changes. In the global forecasting stage, the difference between the SSA-GRU and ITSSA-GRU models is relatively small; the RMSE of ITSSA-GRU is only improved by 6.92% compared with the SSA-GRU model, while the range value is only improved by 1.35%. There is a particularly noteworthy phenomenon in that the four neural network models have a short period of error surge in the middle and late stages of forecasting, which may be related to the poor frequency stability of GEO type satellites.

**Figure 19:**
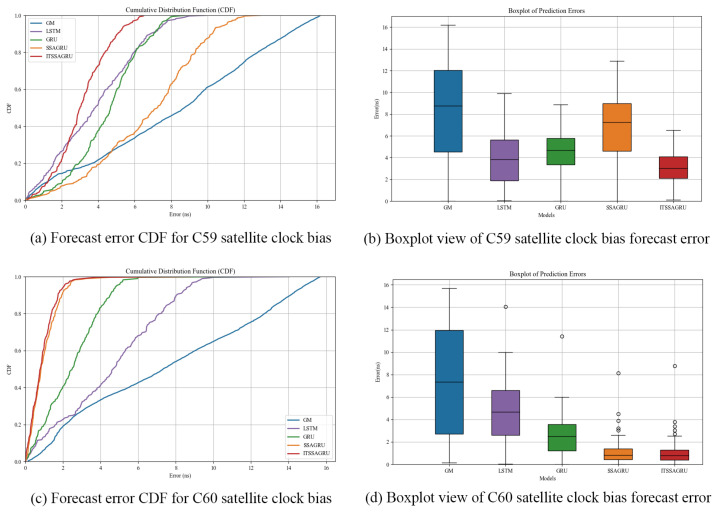
CDF and box plot analysis of satellite clock bias forecast for satellites C59 and C60.

Figure 19 shows the forecasting error CDF plots and box plots for satellites C59 and C60. From Figure 19a,c, it can be seen that there is a major difference in the trend of the error forecast CDF plots for satellites C59 and C60 in comparison to that of the four MEO and IGSO type satellites. The steeper trend of the CDF plots becomes flatter for all five models, indicating that the forecasting accuracy of all five models declines somewhat for GEO orbits. Although the forecasting errors of each model have some shortcomings compared with the experimental results for the other two orbit types, the CDF plots of the ITSSA-GRU model are steeper in comparison. In particular, the steepening trend is more obvious for satellite C59. In the results for satellite C60, the steepening trends of the CDF plots of the ITSSA-GRU and SSA-GRU models are similar, with the former being slightly steeper. Figure 19b shows the error box plots for satellite C59, from which it can be clearly seen that the ITSSA-GRU model has an obvious advantage in forecasting accuracy compared to the other three models; it has a shorter box, and the median and quartile positions are obviously lower, indicating that the ITSSA-GRU model has significantly better forecasting ability. Figure 19d shows the error box plots for satellite C60, from which it can be seen that the CDF plots for this satellite have a similar trend in terms of their overall steepness. The difference between the box size and the median and quartile positions is not large, and the forecasting effects of the SSA-GRU and ITSSA-GRU models are very close to each other.

### 4.4. Discussion

The experimental results show that the GM(1,1) model performs outstandingly in the pre-prediction stage and effectively avoids the problem of large initial prediction errors. However, with prolongation of the forecasting time the predictive ability of GM(1,1) becomes relatively limited, especially in long-term forecasting, and the phenomenon of error accumulation becomes more obvious. In contrast, the neural network models show better prediction performance. However, the forecast results for GEO satellite are relatively poor compared with those for MEO and IGSO satellites. This is manifested in the problems of large error fluctuation amplitudes and obvious fluctuation, which may be related to the existence of the obvious phase jump phenomenon in GEO satellites.

The ITSSA-GRU model demonstrates excellent performance in clock bias forecasting experiments for satellites with different types of orbits as well as for satellites carrying different types of atomic clocks. The ITSSA-GRU model demonstrate superior results in different forecast time periods compared to the other four models, indicating that the proposed model has excellent generalizability and forecasting ability. In particular, the forecasting accuracy and stability of the ITSSA-GRU model are significantly improved in the middle and late forecast stages, highlighting its excellent performance over longer time spans. Therefore, the validity and feasibility of the ITSSA-GRU model for predicting BDS clock bias are proven.

According to the comprehensive analysis of the experimental results, the GRU model performs better than the LSTM model in the clock bias forecasting task in general. Although the forecasting accuracy of the LSTM model was slightly better than that of the GRU model in the C40 satellite forecasting experiment, the GRU model showed better forecasting effect in all other experiments. These results strongly confirm the advantage of the simpler GRU model with fewer parameters in the clock bias forecasting task. Furthermore, they demonstrate the excellent ability of GRUs to manage the long-term dependence of time series data, and highlight the importance of a simplified model structure to realizing improved prediction performance in specific satellite clock prediction scenarios.

In our experiments, the SSA-GRU model improved upon the GRU model after the SSA was used to optimize the parameters of the GRU. Using the SSA to optimize the parameters of the GRU can reduce the sensitivity of the model due its hyperparameter settings, and can prevent the GRU becoming stuck in local optima, thereby improving the generalization ability and forecasting results of the model. Our experimental results show that the SSA-GRU model can effectively improve the prediction accuracy of clock bias, especially in the late prediction period, which can reduce the growth trend of the error, proving the effectiveness of using the SSA in combination with the GRU for the experimental modeling of satellite clock bias forecasting. However, the SSA-GRU has obvious problems as well. For example, in the experiment with the C37 satellite, the forecasting accuracy of the SSA-GRU model in the early stage was not high, and was even inferior to that of the LSTM and GRU models, which did not undergo parameter optimization. In addition, in the experiment with the C40 satellite, although the SSA-GRU performed excellently in the early and middle stages, its forecasting error was significantly larger than that of the other three neural network models after 12 h. In the later stage, the forecast result was obviously poor. Although optimizing the parameters of the GRU using the SSA can improve forecasting ability to a certain extent, the improvement is not stable, as the SSA continues to risk falling into local optimal solutions, especially in complex high-dimensional spaces.

After analyzing and researching the SSA and methods for its improvement, this paper uses two methods to improve the sparrow search method and achieve significant optimization results: iterative chaos mapping and t-distribution variation. Iterative chaos mapping improves the way in which the SSA initializes the population in order to generate an initial value with greater randomness, which increases the diversity of the population and avoids the algorithm prematurely falling into a local optimal solution. The t-distribution variation modifies the strategy used for iterative updating the individuals, which effectively enhances the algorithm’s searching ability, improves its robustness, and reduces the risk of falling into a local optimal solution. The ITSSA algorithm proposed in this paper improves on the SSA, with stronger search ability compared with the traditional SSA, and the experimental results prove the effectiveness of the two optimization methods in improving the SSA. Our results show that the ITSSA-GRU model corrects the problems of insufficient accuracy in the early stage and large fluctuation of the error range in the later stage that can appear in the SSA-GRU model while further improving the prediction accuracy of the SSA-GRU model. Compared with the other four models, the ITSSA-GRU model maintains higher forecasting accuracy and a smaller error range.

## 5. Conclusions

Traditional satellite clock bias forecasting models lack sufficient nonlinear expression ability, resulting in error accumulation over time and ineffective prediction of clock bias in the medium and long term. While neural network models can address nonlinearity, their performance varies and they are highly sensitive to hyperparameters, impacting their forecasting accuracy. To enhance BDS-3 satellite clock bias forecasting we propose the ITSSA-GRU model, which integrates SSA enhancements and addresses the slow convergence and hyperparameter sensitivity of GRUs. We compare our proposed ITSSA-GRU model experimentally with the LSTM, GRU, SSA-GRU, and GM(1,1) models, demonstrating its superior forecasting accuracy in the short, and medium, and long term along with its stability across different satellites and orbits. The results of this study underscore the potential of ITSSA-GRU’s potential to improve GNSS accuracy.

In addition, this paper mainly focuses on the BDS-3 satellite, due to the fact that satellites carrying the same atomic clocks under different orbits were selected as research objects in order to facilitate a comparison of satellites with different orbit types and prove the generalization ability of the model. Thus, the influence of different atomic clock types on the forecasting effect of the ITSSA-GRU model under the same orbit type needs further in-depth study at a later stage.

## Figures and Tables

**Table 1:** ITSSA-GRU model parameters and thresholds.

Hyperparameters	Parameters or Thresholds
num_layers	2
step_size	30
hidden_size	10–40
num_epochs	500–2000
learning_rate	0.001–0.1
producers_percentag	20%
vigilantes_percentag	10%
search_epochs	100

**Table 2:** Percentage increase in the MEO satellite forecast error.

Forecast Time/h	Algorithm Name	Improvement Percentage of C36 Satellite		Improvement Percentage of C37 Satellite	
		**RMSE**	**Range**	**RMSE**	**Range**
3	GM(1,1)	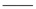	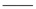	41.14%	49.01%
	LSTM	29.61%	32.32%	27.91%	18.35%
	GRU	28.92%	15.90%	1.06%	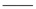
	SSA-GRU	18.54%	2.43%	22.60%	3.01%
6	GM(1,1)	37.43%	46.97%	74.56%	71.14%
	LSTM	54.85%	45.53%	36.84%	18.35%
	GRU	49.32%	33.22%	25.77%	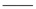
	SSA-GRU	45.89%	22.99%	38.46%	9.15%
12	GM(1,1)	79.30%	77.77%	89.36%	85.20%
	LSTM	49.25%	45.53%	53.78%	27.93%
	GRU	40.94%	33.22%	60.43%	44.87%
	SSA-GRU	39.88%	22.99%	62.07%	37.68%
24	GM(1,1)	87.92%	89.37%	89.36%	85.80%
	LSTM	45.05%	52.93%	64.44%	58.48%
	GRU	36.46%	41.57%	60.85%	48.20%
	SSA-GRU	39.60%	35.78%	37.27%	5.74%

**Table 3:** Percentage increase in the IGSO satellite forecasting error.

Forecast Time/h	Algorithm Name	Improvement Percentage of C38 Satellite		Improvement Percentage of C40 Satellite	
		**RMSE**	**Range**	**RMSE**	**Range**
3	GM(1,1)	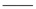	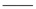	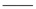	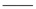
	LSTM	32.42%	34.85%	5.76%	0.66%
	GRU	13.50%	24.24%	2.60%	2.27%
	SSA-GRU	21.72%	26.20%	0.38%	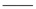
6	GM(1,1)	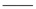	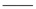	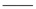	11.73%
	LSTM	41.24%	47.30%	4.43%	0.66%
	GRU	20.09%	33.66%	9.12%	11.73%
	SSA-GRU	22.27%	24.35%	4.07%	6.23%
12	GM(1,1)	63.08%	66.56%	52.12%	63.65%
	LSTM	51.34%	47.30%	2.71%	0.66%
	GRU	22.09%	33.66%	7.33%	11.73%
	SSA-GRU	25.29%	24.35%	7.73%	6.23%
24	GM(1,1)	86.25%	84.40%	83.85%	81.40%
	LSTM	46.03%	47.30%	9.83%	0.66%
	GRU	39.29%	33.66%	10.34%	11.73%
	SSA-GRU	25.00%	24.35%	23.15%	6.23%

**Table 4:** Percentage increase in the GEO satellite forecasting error.

Forecast Time/h	Algorithm Name	Improvement Percentage of C59 Satellite		Improvement Percentage of C60 Satellite	
		**RMSE**	**Range**	**RMSE**	**Range**
3	GM(1,1)	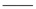	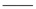	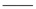	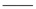
	LSTM	75.59%	66.16%	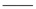	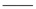
	GRU	28.57%	50.85%	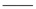	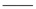
	SSA-GRU	34.87%	42.54%	11.38%	2.58%
6	GM(1,1)	8.20%	8.50%	26.22%	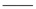
	LSTM	63.52%	58.65%	33.52%	17.69%
	GRU	23.55%	40.90%	12.32%	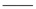
	SSA-GRU	36.00%	46.40%	1.63%	2.58%
12	GM(1,1)	42.60%	28.10%	70.05%	48.50%
	LSTM	42.38%	36.60%	60.62%	36.53%
	GRU	24.42%	16.29%	37.16%	6.45%
	SSA-GRU	42.38%	34.82%	3.36%	2.58%
24	GM(1,1)	63.89%	59.75%	86.36%	48.95%
	LSTM	23.49%	34.07%	77.21%	42.91%
	GRU	30.06%	26.58%	58.70%	29.56%
	SSA-GRU	53.28%	49.34%	6.92%	1.35%

## Data Availability

The model code can be provided upon request from the corresponding author, Feng Liu. Data are available from the International GNSS Monitoring and Assessment System (iGMAS) at http://www.igmas.org (accessed on 5 July 2023).
